# A randomised, multicentre clinical trial of specialised palliative care plus standard treatment versus standard treatment alone for cancer patients with palliative care needs: the Danish palliative care trial (DanPaCT) protocol

**DOI:** 10.1186/1472-684X-12-37

**Published:** 2013-10-24

**Authors:** Anna T Johnsen, Anette Damkier, Tove B Vejlgaard, Jane Lindschou, Per Sjøgren, Christian Gluud, Mette A Neergaard, Morten Aa Petersen, Lena E Lundorff, Lise Pedersen, Peter Fayers, Annette S Strömgren, Irene J Higginson, Mogens Groenvold

**Affiliations:** 1 Department of Palliative Medicine, The Research Unit, Bispebjerg Hospital 20D, Bispebjerg Bakke 23, DK-2400 Copenhagen, NV,Denmark; 2 Palliative Team Fyn, Odense University Hospital, Odense, Denmark; 3 Palliative Team Vejle, Vejle Hospital, Vejle, Denmark; 4 Copenhagen Trial Unit, Centre for Clinical Intervention Research, Rigshospitalet, Copenhagen University Hospital, Copenhagen, Denmark; 5 Department of Oncology, Section of Palliative Medicine, Rigshospitalet, Copenhagen University Hospital, Copenhagen, Denmark; 6 The Palliative Team, Aarhus University Hospital, Aarhus, Denmark; 7 Palliative Team Herning, Herning Hospital, Herning, Denmark; 8 Institute of Applied Health Sciences, University of Aberdeen Medical School, Foresterhill, Aberdeen, UK; 9 Department of Oncology, Rigshospitalet, Copenhagen University Hospital, Copenhagen, Denmark; 10 King’s College London, Cicely Saunders Institute, Department of Palliative Care, Policy and Rehabilitation, London, UK; 11 Institute of Public Health, University of Copenhagen, Copenhagen, Denmark

**Keywords:** Palliative care, End-of-life care, Advanced cancer, Randomised clinical trial, Quality of life, Needs assessment, Patient satisfaction, Cost-effectiveness, Study protocol

## Abstract

**Background:**

Advanced cancer patients experience considerable symptoms, problems, and needs. Early referral of these patients to specialised palliative care (SPC) could improve their symptoms and problems.

The Danish Palliative Care Trial (DanPaCT) investigates whether patients with metastatic cancer, who report palliative needs in a screening, will benefit from being referred to ‘early SPC’.

**Methods/Design:**

DanPaCT is a clinical, multicentre, parallel-group superiority trial with balanced randomisation (1:1). The planned sample size is 300 patients. Patients are randomised to specialised palliative care (SPC) plus standard treatment versus standard treatment. Consecutive patients from oncology departments are screened for palliative needs with a questionnaire if they: a) have metastatic cancer; b) are 18 years or above; and c) have no prior contact with SPC. Patients with palliative needs (i.e. symptoms/problems exceeding a certain threshold) according to the questionnaire are eligible. The primary outcome is the change in the patients’ primary need (the most severe symptom/problem measured with the European Organisation for Research and Treatment of Cancer Quality of Life Questionnaire (EORTC QLQ-C30)). Secondary outcomes are other symptoms/problems (EORTC QLQ-C30), satisfaction with health care (FAMCARE P-16), anxiety and depression (the Hospital Anxiety and Depression scale), survival, and health care costs.

**Discussion:**

Only few trials have investigated the effects of SPC. To our knowledge DanPaCT is the first trial to investigate screening based ‘early SPC’ for patients with a broad spectrum of cancer diagnosis.

**Trial registration:**

Current controlled Trials NCT01348048

## Background

The aim of palliative care is to relieve suffering and improve quality of life in patients with a life-threatening disease [1]. Palliative care can be divided into basic palliative care provided by general practitioners, home-care services, and hospitals, and specialised palliative care (SPC) which is offered by palliative care teams, departments of palliative medicine, and hospices [2].

In 2011, 38% of patients dying from cancer in Denmark had been in contact with SPC [2]. Their median survival from first contact with SPC was six weeks [2]. Our recent, nation-wide study showed that large proportions of advanced cancer patients not in contact with SPC had symptoms and problems such as pain, fatigue, and depression [3,4]. One possible solution to the inadequate management of symptoms could be the referral of these patients to SPC at an earlier time in their disease trajectory (‘early SPC’).

A recent randomised trial investigated ‘early SPC’ plus standard treatment versus standard treatment alone [5-7]. It showed that patients newly diagnosed with metastatic lung cancer who were randomised to SPC plus standard treatment obtained better quality of life, less depression, and longer survival than patients receiving standard treatment. Thus, the trial provided preliminary evidence that ‘early SPC’ seem beneficial in lung cancer. However, this was a single-centre U.S.A. trial assessing a small and selected group of patients. We wanted to assess the effects of ‘early SPC’ in a Danish setting and in a population with various cancer diagnoses. Furthermore, not all patients with metastatic cancer need SPC and therefore including patients with no palliative needs may ‘dilute’ possible positive effects of ‘early SPC’. Accordingly, we therefore want to investigate whether patients with metastatic cancer who report palliative needs (symptoms and problems) in a screening will benefit from being referred to SPC.

The aim of the present paper is to describe the Danish Palliative Care Trial (DanPaCT) protocol.

## Methods and design

### Trial design

The trial is a clinical, multicentre, parallel-group superiority trial with balanced randomisation (1:1) conducted at six Danish SPC centres. The protocol has been approved by the local regional ethics committee (the Ethics Committee for the Capital Region, Denmark; journal number H-3-2010-144) and registered at www.clinicaltrials.gov (NCT01348048).

#### Setting

Patients from departments of oncology are randomised to SPC plus standard care versus standard care. Patients are recruited at the following hospitals: Copenhagen University Hospital (Rigshospitalet), Aarhus University Hospital, Odense University Hospital, Herning Hospital, and Vejle Hospital. Information about the six SPC centres can be seen in Table 1.

**Table 1:** Description of the sixspecialised palliative care centres performing the intervention in the DanPaCT trial (2013 data)

	**Multi-professional team**	**Organisation**	**Number of patients treated peryear**	**Year of establishment**
Department of Palliative Medicine, Bispebjerg Hospital	Doctors, nurses, physiotherapist, psychologists, social worker, chaplain, secretary, and volunteers.	Home-visits (nursing homes included), 12 hospital beds, out-patients’ clinic, consultation in other hospital departments.	About 400	1997
Section of Acute Pain Management and Palliative Medicine, Rigshospitalet	Doctors, nurses, psychologists, and social worker.	Home-visits (nursing homes included), out-patients’ clinic, consultations in other hospital departments.	About 100	2012
The Palliative Team, Aarhus University Hospital	Doctors, nurses, physiotherapist, psychologist, social worker, chaplain, and volunteers.	Home-visits (nursing homes included), three hospital beds, out-patients’ clinic, consultations in other hospital departments.	About 380	1999
Palliative Team Funen, Odense University Hospital	Doctors, nurses, physiotherapist, psychologist, social worker, chaplain, and volunteers.	Home-visits (nursing homes included), out-patients’ clinic, consultations in other hospital departments.	About 300	2004
Palliative Team Vejle, Vejle Hospital	Doctors, nurses, physiotherapist and psychologist.	Home-visits (nursing homes included), out-patients’ clinic, consultations in other hospital departments.	About 250	2005
Palliative Team Herning, Herning Hospital	Doctors, nurses, physiotherapist, psychologist and pharmacist.	Home-visits (nursing homes included), three hospital beds, out-patients’ clinic, consultations in other hospital departments.	About 230	2002

### Patient inclusion and exclusion criteria

Patients in contact with the oncology departments who have cancer stage four according to the ‘TNM’ (TNM stands for Tumor, Node, Metastases) classification [8] or cancer in the central nervous system grade three or four, are at least 18 years, live in the area of one of the participating SPC centres, and who have not had contact with an SPC during the previous year receive a screening questionnaire. If, according to their answers in the questionnaire, they have a palliative need and four additional symptoms (see definition below) they are informed about the trial and invited to participate. Patients who provide informed consent are randomised. Patients are excluded from the trial if they cannot understand Danish well enough to fill in a questionnaire or are considered incapable of complying with the trial protocol.

### Screening: assessment of palliative needs and additional symptoms

Patients are screened with the European Organisation for Research and Treatment of Cancer Quality of Life Questionnaire (EORTC QLQ-C30) [9].

The EORTC QLQ-C30 [9] assesses health-related quality of life and consists of nine multi-item scales measuring: physical function, role function, emotional function, cognitive function, social functioning, global health status/quality of life, fatigue, nausea and vomiting, and pain, and six single-item scales: dyspnoea, insomnia, lack of appetite, constipation, diarrhoea and financial difficulties.

Patients are defined as having a palliative need and four additional symptoms (and are thus eligible for the trial) if they:

● Score at least 50% of the score corresponding to maximal symptom burden or maximally reduced functioning on at least one of the following scales in EORTC QLQ-C30: physical function, role function, emotional function, nausea and vomiting, pain, dyspnoea, or lack of appetite; AND

● Have four additional symptoms (defined as EORTC QLQ-C30 scale score of at least 33% of the score corresponding to maximal symptom burden or maximally reduced functioning) out of the 14 EORTC QLQ-C30 scales (global health status/quality of life excluded).

### Randomisation

Central randomisation via telephone is carried out by the Copenhagen Trial Unit (CTU), which is independent of the trial administration office. The allocation sequence is computer-generated 1:1 with varying block size and is kept unknown for all investigators. Randomisation is stratified by the variable ’primary need’ (see description in the section ‘Outcomes’).

### Assessments and questionnaires

**Figure 1:**
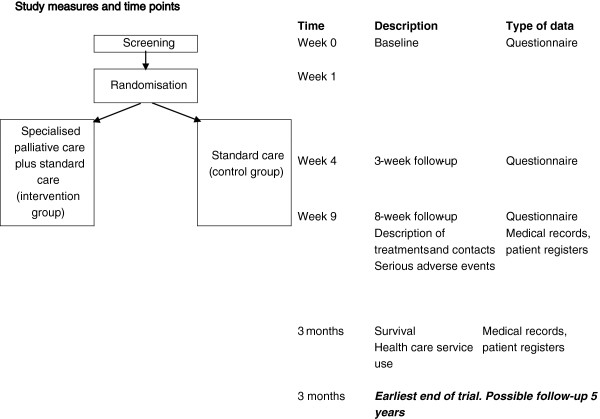
Study measures and time points.

All randomised patients are assessed with a questionnaire (see Figure 1): a) at baseline (the screening); b) after a 3-week follow-up period; and c) after an 8-week follow-up period.

The questionnaires include EORTC QLQ-C30 (described in the section on ‘screening’), the FAMCARE-p16 questionnaire [10] and the Hospital Anxiety and Depression Scale (HAD scale) [11]. FAMCARE-p16 assesses advanced cancer patients’ satisfaction with the health care system. The questionnaire includes several items that address key areas of palliative care. It uses 5-point Likert scales where 1=‘very satisfied’, 2=‘satisfied’, 3=‘undecided’, 4=‘dissatisfied’ and 5=‘very dissatisfied’. The patients are asked to rate the care they received within the previous month. In addition four items measuring satisfaction were developed for this particular trial.

The HAD [11] scale assesses anxiety and depression and was developed for people with a somatic disease. The questionnaire has been used in many studies of advanced cancer patients, and its validity and reliability has been tested on several occasions with a generally positive conclusion [12].

Finally, we included six additional newly developed items to measure pain, three items measuring appetite loss, and three items about dyspnoea. These items were selected from the item banks of the EORTC Computerised Adaptive Testing Project [13,14] and will be used in sensitivity analysis and subsequent methodological analyses only.

Three months after end of the intervention information about death, use of health services and treatments are retrieved from registers by investigators who are blinded and not aware of treatment allocation.

Clinical data is extracted from the medical records regarding primary tumour, sex, age, stage of cancer according to the TNM system, time of primary diagnosis, and treatment status.

To describe the interventions given and the ‘palliative activity’ in both groups all treatments and contacts with the health care system are registered. This is done by reviewing the medical records from the time of randomisation until the 8-week follow-up.

### Outcomes

*The primary outcome* is estimated as the difference between the intervention and the control group in the change from baseline to the weighted mean of the 3- and 8-week follow-up measured as area under the curve (AUC) for the EORTC QLQ-C30 scale score that constitutes the primary need. The *primary need* is defined as the palliative need having the highest intensity at baseline according to the EORTC QLQ-C30. Seven different palliative needs (listed in the section on screening) are considered when defining the primary need.

*Secondary outcomes*, estimated in the same way, are a) the remaining symptoms and problems measured by the EORTC QLQ-C30 (14 scales), b) anxiety and depression measured by the HAD Scale c) the patients’ evaluation of treatment and care provided by the health care system measured by the FAMCARE-p16, d) survival, and e) economical consequences per week from the start of the trial to minimum three months after the end of the intervention.

### Sample size estimation

We know from previous studies (unpublished data) that the standard deviation (SD) for a difference between repeated measurements (three weeks apart) in the EORTC QLQ-C30 is 15 to 22 points. Therefore, we assume that the primary outcome has an SD of 20 points in the present trial. We wish to be able to detect a difference of 7.5 points (a difference between 5 and 10 is normally judged clinically significant [15]). With a risk of type I error of 0.05 and type II error of 0.10 we need 150 patients in each intervention group, i.e., a total of 300 patients, and 50 from each centre.

### Plan of analysis

Analyses will be made using SAS statistical software version 9 [16]. The primary analysis will be based on the intention-to-treat principle. If there are more than 5% missing answers we will use multiple imputations with the following variables in our model: age, sex, diagnosis, cancer stage, and The World Health Organisations(WHO) performance score.

Analyses will be made as multiple regressions. The primary analysis of all outcomes will be adjusted for the stratification variable, the patient’s ‘primary need’. Sensitivity analyses will further be adjusted for WHO performance status, and ‘centre’. If there is an imbalance in the two treatments groups we shall further consider adjusting for gender, age, cancer stage, diagnosis, co-morbidity, treatment status and education which may have a prognostic value [17,18].

#### Blinding

Allocation status cannot be blinded for the participants and trial personnel. However, concealment of allocation is carefully maintained during retrieval of outcome information from national registers. Further, all statistical analyses will be carried out blinded to intervention with the two groups coded as, e.g., A and B. Two conclusions will be drawn, one assuming A is the experimental group and B is the control group, and one assuming the opposite. After this, the blind will be broken.

### Ethics

The justification for carrying out the trial is the sparse evidence concerning the beneficial and harmful effects of ‘early SPC’. In addition, patients randomised to standard treatment in this trial receive the same treatment as they would have had it not been for this trial. Some of these patients may seek referral to SPC and of course, this will be registered but not prevented.

## Discussion

Few randomised clinical trials have investigated the effect of SPC and thus, the evidence regarding SPC is sparse [5,19-29]. DanPaCT is a randomised multicentre trial including patients with advanced cancers from multiple organs. To our knowledge it is the first trial to investigate ‘early SPC’ for patients with a broad spectrum of cancer diagnosis, and the first trial to investigate screening-based referral to SPC. In addition it is the first to provide detailed information about the specific interventions given by the SPC centres; a knowledge that has been requested [30].

In DanPaCT, the primary outcome is tailored to the patient by being the patient’s most pronounced symptom/problem (‘primary need’). For some patients, the primary outcome is pain, for others it is appetite loss. The strength of this approach is that the primary outcome is relevant for all patients, and that it takes into account the multidimensionality of palliative care without summing up the different symptoms/problems, which may lead to dilution of the measurement of effect.

The treatment allocation cannot be blinded for participants and staff, which is of course a limitation [31,32]. This lack of blinding of the treatment after allocation may have several consequences. It is possible that patients in the SPC group will receive less attention from the oncology departments. To determine whether this is the case, the two groups’ number of contacts with the oncology departments will be assessed and compared. Although we consider it unlikely, it is also possible that patients in the SPC group will receive a better SPC treatment than patients receiving usual care, and it is possible that patients in the SPC group will underreport their symptoms at follow-up. However, the patients are not informed about the primary outcome of the trial. Further, assessment of survival and health economics will be based on registry data and hence, blinded to intervention. In addition, the randomisation will be performed centrally and allocation carefully concealed to the project nurses in order to avoid selection bias, and data-analysts will be blinded to intervention [31,32].

The trial does not include all relevant palliative needs (e.g., existential and spiritual needs). In addition, patients with metastatic cancer constitute a heterogeneous group, and SPC may work better for patients with some primary diagnoses than for others, and this could weaken the power of our trial. It is also possible that the various SPC centres are better at treating different symptoms, and that a high effect from one SPC centre is weakened by the others. However, the heterogeneity of patients and centres increase the generalisability of our results.

When the trial started we had different inclusion criteria from what has been presented here. Originally, the patients needed to have two palliative needs instead of only one, and the definition of palliative need was originally stricter (in order to have a palliative need the patients had to consider the symptom to be a problem and have a need for help with the symptom). However, after the first two months of the trial, we realised that our initial inclusion criteria was too strict. Out of the first 168 patients completing a screening questionnaire, only eight were randomised. Therefore we changed the inclusion criteria in order to make a larger proportion of patients eligible. It is the revised and final inclusion criteria which are presented in this paper. The change was approved by the local regional ethics committee, the funding body and reported to clinicaltrials.gov.

## Conclusion

The DanPaCT trial aims at investigating whether patients with metastatic cancer who report palliative needs in a screening will benefit from being referred to ‘early SPC’. We also study the economical and survival consequences of such a referral.

## Competing interests

The authors declare that they have no competing interests.

## Authors’ contributions

ATJ, AD, TBV, JL, PS, CG, MAN, MAP, LEL, LP, PF, IJH, MG took part in designing the trial. JL, CG, MAP, PF with special competence in RCT and/or statistical analysis, and ATJ, AD, TBV, PS, MAN, LEL, LP, IJH, MG with special competence in palliative care. AD, TBV, PS, MAN, LEL, LP, MG are clinical investigators and in charge of the data collection, and ASS helped collect data. ATJ was project coordinator and MG received the funding for the study. ATJ drafted the paper and all authors read, amended and approved the final manuscript.

## Pre-publication history

The pre-publication history for this paper can be accessed here:

http://www.biomedcentral.com/1472-684X/12/37/prepub
